# IgG4-related tubulointerstitial nephritis accompanied with cystic formation

**DOI:** 10.1186/1471-2490-14-54

**Published:** 2014-07-20

**Authors:** Hideo Fukuhara, Yoshinori Taniguchi, Manabu Matsumoto, Naoto Kuroda, Satoshi Fukata, Keiji Inoue, Shimpei Fujimoto, Yoshio Terada, Taro Shuin

**Affiliations:** 1 Department of Urology, Kochi Medical School, Kohasu, Oko, Nankoku, Kochi 783-8505, Japan; 2 Department of Endocrinology, Metabolism and Nephrology, Kochi, Japan; 3 Department of Pathology, Kochi Medical School, Kochi, Japan; 4 Department of Diagnostic Pathology, Kochi Red Cross Hospital, Kochi, Japan

**Keywords:** IgG4-related tubulointerstitial nephritis, Renal cyst change, Collecting duct

## Abstract

**Background:**

An immunoglobulin G4 (IgG4)-related disease is important disease in differential diagnosis of tumors in kidney, pancreas, lung and other organs. The imaging findings of IgG4-related kidney diseases are usually expressed as defect contrast region, while cystic formation in kidney is extremely rare. Here, we report a case of IgG4-related tubulointerstitial nephritis with renal cystic change caused by the narrowing or obstruction of collecting duct in renal medulla.

**Case presentation:**

Abdominal contrasted CT scan showed a 31 × 24 mm cystic tumor at the upper pole of the right kidney and multiple low-attenuation areas in the left kidney. ^18^ F-fluorodeoxyglucose (FDG)-PET/CT scan showed moderate FDG accumulation of cystic tumor in marginal lesion. In addition, FDG-PET/CT scan also showed moderate FDG accumulation in the pancreatic body. Laparoscopic right nephrectomy was performed. Histological examination was revealed lymphoplasmacytic infiltrate with focal fibrosis and severe narrowing or obstruction of lumen of collecting duct in renal medulla. Furthermore, the IgG4 positive plasma cells infiltrated exceeding 10 cells per one high-power field in renal medulla. The ratio of IgG4-plasma cells to IgG-positive plasma cells was about 50%. The serum level of IgG4 was also elevated (218 mg/dl). Based on these findings, we finally diagnosed IgG4-related tubulointerstitial nephritis with renal cystic change.

**Conclusion:**

IgG4-related kidney disease might cause cystic formation by severe narrowing and obstruction of collecting duct.

## Background

An immunoglobulin G4 (IgG4)-related disease is a newly-proposed clinical disease entity characterized by elevated serum IgG4 and IgG4 positive plasma cell infiltration in various organs. IgG4-related disease was first described as autoimmune pancreatitis (AIP) and has subsequently been described in other organs [1-3]. The affected common site is considered to be pancreas, liver, salivary gland, lung, breast, prostate and kidney. The histological characteristics are lymphoplasmacytic infiltrate, IgG4 plasma cell and fibrosis [4]. Imaging feature is often described as an interstitial lesion [5]. However, we sometimes encounter pseudotumor formation which is not easy to distinguish from malignant tumor [6,7]. Herein, we report a case of IgG4-related tubulointerstitial nephritis with renal cystic change caused by the narrowing or obstruction of collecting duct in renal medulla.

## Case presentation

**Figure 1:**
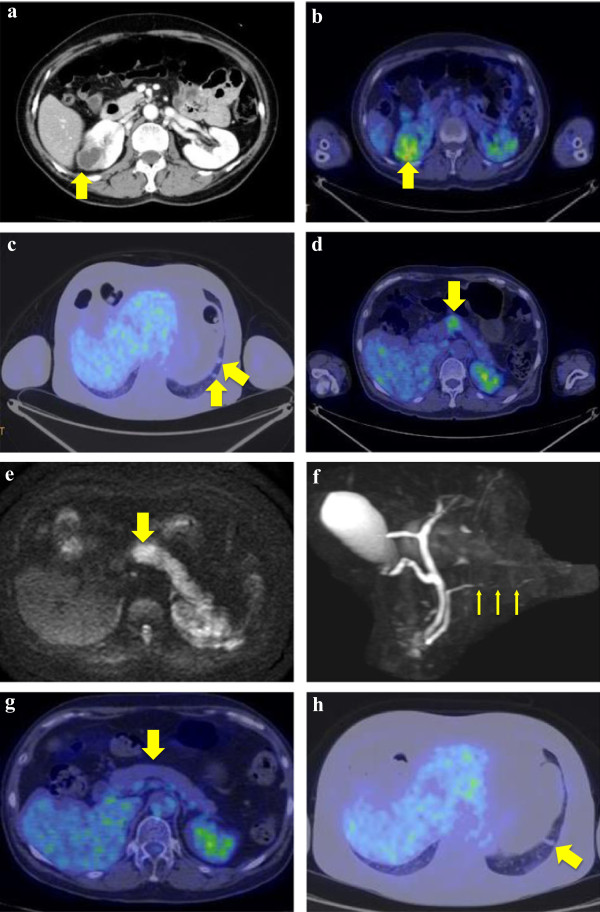
**Imaging findings. a.** Abdominal computed tomography scan shows 31×24 mm cystic lesion in right kidney (arrows). **b.** PET/CT scan showed moderate FDG accumulation of cystic lesion in right kidney (arrows). **c.** PET/CT scan showed moderate FDG accumulation of lung tumor (arrows). **d.** PET/CT scan showed moderate FDG accumulation of pancreatic body (arrows). **e.** Diffusion-weighted MRI showed high intensity area in pancreatic body (arrows). **f.** MRCP showed irregular narrowing in main pancreatic duct (arrows). **g.** PET/CT scan demonstrated a decrease of FDG accumulation in pancreatic body (arrows). **h.** PET/CT scan demonstrated a decrease of FDG accumulation in lung tumor (arrows).

A 63-year-old woman was referred to Kochi University Hospital with a renal tumor discovered by medical examination, incidentally discovered on a computed tomography (CT) scan. There was no previous medical history and family history of kidney disease. Her vital signs were normal value. Blood electrolytes, proteinogram, renal function and hepatic enzymes showed all normal value. Soluble IL-2 receptor was slightly elevated (703 U/ml (normal, 145–519)). The other tumor markers were all within the normal range. Abdominal contrasted CT scan showed a 31 × 24 mm cystic tumor at the upper pole of the right kidney and multiple low-attenuation areas in the left kidney (Figure 1a).

**Figure 2:**
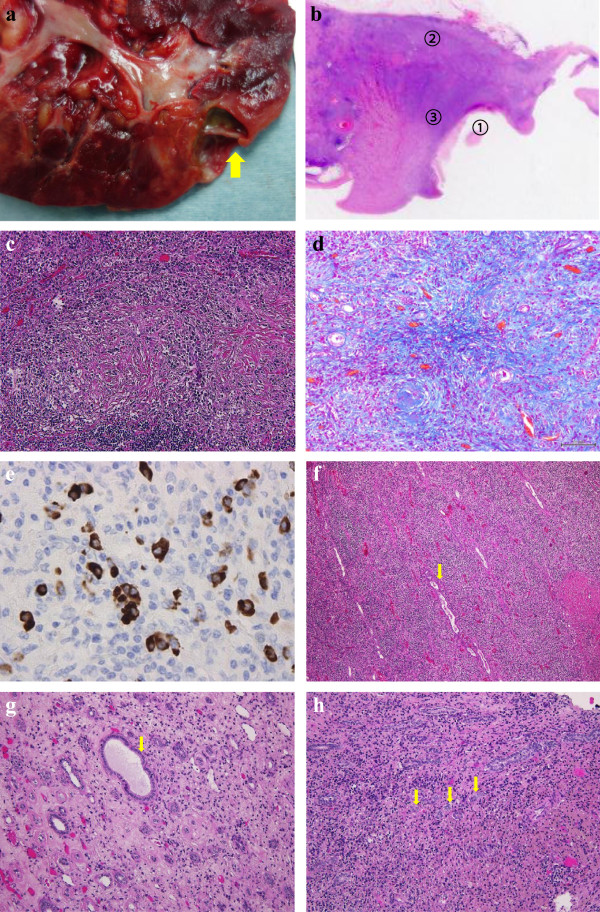
**Macroscopic and microscopic findings. a.** The macroscopic findings of renal cystic tumor (arrows). **b.** low power loupe images of 3 parts (①; cystic cavity, ②; renal cortex, ③; renal medulla). **c.** H&E stain. Marked lymphoplasmacytic infiltrate and storiform fibrosis. **d.** Storiform fibrosis with Azan staining **e.** Immunohistochemical result. A significant amount of IgG4-positive plasma cells infiltrates in renal medulla. **f.** Collecting duct was compressed longitudinary in renal cortex (arows). **g.** Collecting duct was dilated in renal in renal medulla (arrows). **h.** Collecting duct became narrowed and obstructed in renal medulla (arrows).

**Figure 3:**
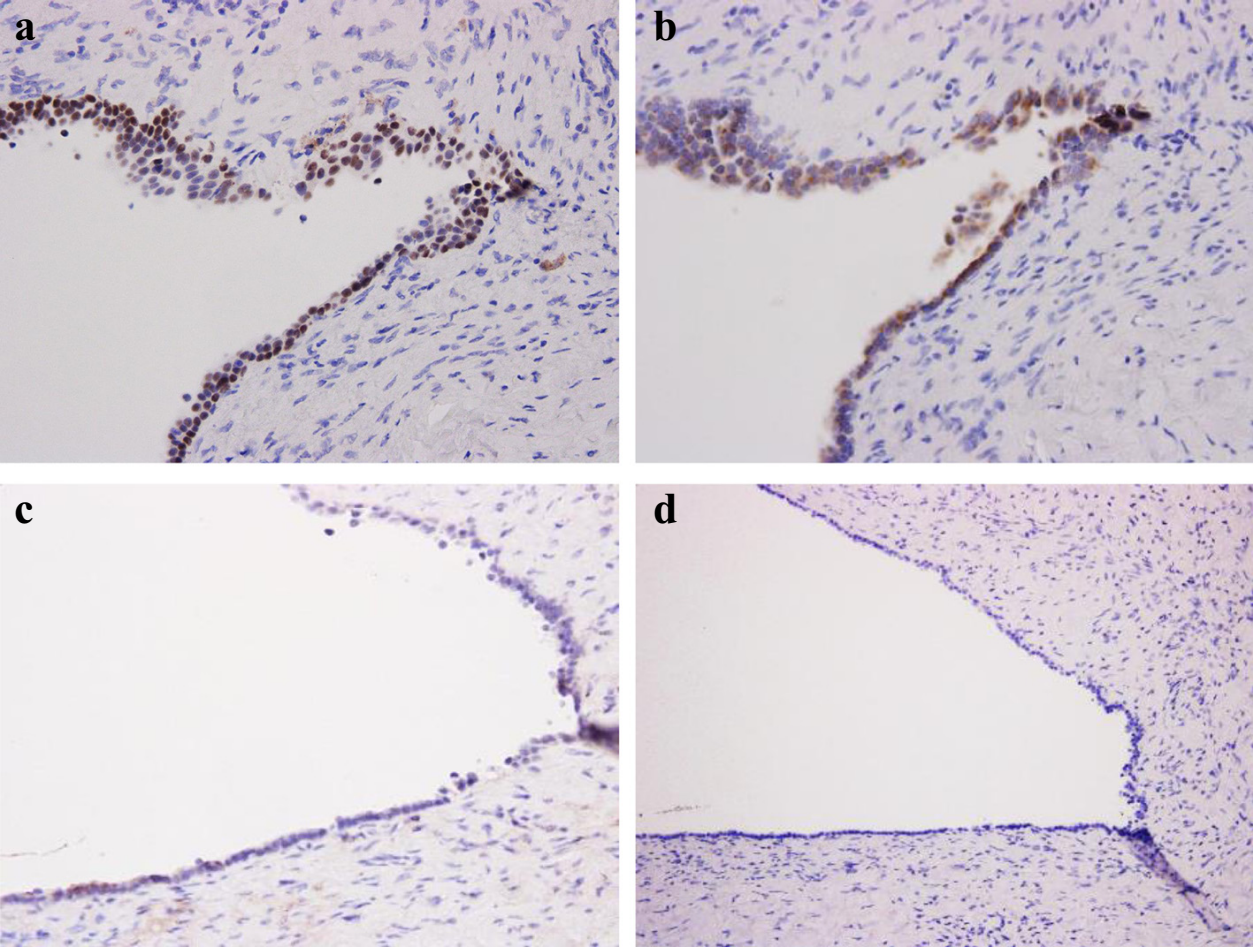
**Immunostaining findings. a.** Immunohistochemical result. PAX2 highlights cells lining in the cyst wall (collecting duct maker). **b.** EMA highlights cells lining in the cyst wall (collecting duct maker). **c.** RCC-Ma negative cells lining in the cyst wall (proximal duct maker). **d.** CD10 negative cells lining in the cyst wall (proximal duct marker).

^18^ F-fluorodeoxyglucose (FDG)-Positron Emission Tomography/Computed Tomography (PET/CT) scan showed moderate FDG accumulation of cystic tumor in marginal lesion (Figure 1b). In addition, FDG-PET/CT scan also showed moderate FDG accumulation in the pancreatic body and small lung mass (Figure 1c, d). Diffusion-weighted magnetic resonance images (MRI) showed high intensity area and focal enlargement on pancreatic body. Furthermore, main pancreatic duct showed irregular narrowing on magnetic resonance cholangio pancreatography (MRCP) (Figure 1e and f), indicating autoimmune pancreatitis.Multiple low-attenuation area in left kidney fitted the imaging findings of tubulointerstitial nephritis. However, right renal cystic tumor had diffuse wall thickening and weak enhancement of the cystic wall on CT scan and renal cell carcinoma with cystic change was suspected. In imaging test, it was very difficult to distinguish from tubulointerstitial nephritis and malignant renal cystic tumor. Ultimately, we made a preoperative diagnosis as suspicious of renal cell carcinoma with cystic change and then performed laparoscopic right nephrectomy. The macroscopic findings of cystic wall were gray white color and no hemorrhage was observed inside (Figure 2a and b). Histological examination was revealed lymphoplasmacytic infiltrate with storiform fibrosis in renal cortex (Azan staining positive) (Figure 2c and d).Immunohistochemically, the IgG4 positive plasma cells infiltrated exceeding 10 cells per one high-power field (Figure 2e). The ratio of IgG4-plasma cells to IgG-positive plasma cells was about 50%.Collecting duct was compressed longitudinally by severe inflammation and fibrosis in renal cortex around cyst (Figure 2f). In contrast, collecting duct had a tendency to dilate in renal medulla adjacent to renal cortex and obstruct in renal medulla away from renal cortex (Figure 2g and h). In additional immunohistochemical analysis of cyst wall showing collecting duct markers, epithelial membrane antigen (EMA), the paired box (PAX) 2 and PAX8, were positive in the lining cell of the cyst wall (Figure 3a and b). While proximal tubule markers, CD10 and renal cell carcinoma marker antigen (RCC-Ma), were negative (Figure 3c and d). Also, cystic wall had no significant malignant components.Based on pathological results after surgery, then we analyzed stored preoperative serum retrospectively. As a result, the patients had hypocomplementemia and polyclonal gammopathy with elevated levels of serum IgG (1934 mg/dl) and IgE (1061 IU/ml). The serum level of IgG4 was also elevated (218 mg/dl). Finally, we diagnosed IgG4-related tubulointerstitial nephritis with renal cystic change according to a diagnostic algorithm of the Japan Society of Nephrology. After the operation, the patient receives steroidal therapy. Oral prednisolone at initial dose of 30 mg/day was administrated after surgery. Six month after therapy, serum level of IgG4 returns to normal level (28.1 mg/dl) and FDG-PET/CT scan showed disappearance of FDG accumulation in pancreatic body and lung mass (Figure 1g and h).

## Discussion

To our knowledge, renal cystic formation of IgG4-related disease in our case is the first reported case in English literatures. The imaging findings of IgG4-related kidney diseases are usually expressed as defect contrast region based mainly on interstitial lesion. While, the mass formation is also found in some cases [6,7]. These cases are difficult to distinguish from renal cell carcinoma by imaging findings. The imaging findings of IgG4-related kidney diseases have some variations. In the representative imaging study by Takahashi et al., they categorized IgG4-related kidney disease into four types (1. mass or nodule; 2. diffuse patchy; 3. kidney swelling; 4. pelvic wall thickening) [8]. This case demonstrated cystic formation and did not correspond to any four types in imaging inspection. Then, cystic type of IgG4-related kidney disease is extremely rare.We assume the mechanism of cystic formation in this case according to pathological examination: lymphoplasmacytic lesion and storiform fibrosis in IgG4-related kidney disease tends to occur in renal cortex. But if inflammation and fibrosis spread to renal medulla, we must consider about affect on collecting duct. In this case, the inflammation and fibrosis in renal cortex spread to renal medulla and induced severe narrowing or obstruction of lumen of collecting duct in renal medulla (Figure 2h). Collecting duct in renal medulla adjacent to renal cortex had a tendency to dilate due to mild inflammation and fibrosis (Figure 2g), whereas collecting duct in renal cortex around cyst was compressed longitudinally by sever inflammation and fibrosis and did not show dilation (Figure 2f).Thus, spread of inflammation and fibrosis from renal cortex induced atrophy and narrowing or obstruction of lumens of collecting duct in renal medulla (Figure 2h). These findings provide that the severe narrowing and obstruction of collecting duct in renal medulla followed by the dilation of proximal site of collecting duct system and finally led to cystic formation in renal cortex. In immunohistochemical analysis of cyst wall showing the cystic change, collecting duct markers, EMA, PAX2 and PAX8, were positive in the lining cell of the cyst wall (Figure 3a and b). While proximal tubule markers, CD10 and RCC-Ma, were negative (Figure 3c and d). Furthermore, the obstruction exists only in collecting duct of renal medulla and there is no adjacent cystic lesion.

One might point that IgG4-related kidney disease occurred with simple renal cyst in this case. However, simple renal cyst generally arises from proximal origin [9] and she has never detected renal abnormality by health check. Therefore, we hypothesize that IgG4-related kidney disease might cause cystic formation by severe narrowing and obstruction of collecting duct.

If this cystic tumor had been diagnosed as an IgG4-related kidney disease by needle biopsy before surgery, we could avoid unnecessary operation and provide steroid therapy. Therefore, it is important to consider a differential diagnosis with IgG4-related disease from malignant cystic disease similar to imaging in this case. Further case investigations on relationship between imaging findings and pathological results should be examined in IgG4-related kidney disease.

## Conclusion

IgG4-related kidney disease might cause cystic formation by severe narrowing and obstruction of collecting duct.

## Consent

Written informed consent was obtained from the patient for publication of this case report and the accompanying images. A copy of written consent is available for review by the Editor-in-Chief of this journal.

## Abbreviations

IgG4: Immunoglobulin G4; AIP: Autoimmune pancreatitis; PAX: Paired box; EMA: Epithelial membrane antigen; RCC-Ma: Renal cell carcinoma marker antigen.

## Competing interests

The authors declare that they have no competing interests.

## Authors’ contributions

HF drafted the report. YT, SF, KI, SF, YT and TS cared for patient and approved the final version of the manuscript. MM and NK performed histopathological examinations. All authors reviewed the report and approved final version of the manuscript.

## Pre-publication history

The pre-publication history for this paper can be accessed here:

http://www.biomedcentral.com/1471-2490/14/54/prepub
